# Understanding implementation barriers in the national scale-up of differentiated ART delivery in Uganda

**DOI:** 10.1186/s12913-020-5069-y

**Published:** 2020-03-17

**Authors:** Henry Zakumumpa, Joseph Rujumba, Japheth Kwiringira, Cordelia Katureebe, Neil Spicer

**Affiliations:** 1 Makerere University, School of Public Health, Kampala, Uganda; 2 Makerere University, School of Medicine, Kampala, Uganda; 3 Department of Sociology, Kyambogo University, Kampala, Uganda; 4 Ministry of Health, AIDS Control Program, Kampala, Uganda; 5 London School of Hygiene and Tropical Medicine, London, UK

**Keywords:** HIV treatment, Health systems, Differentiated service delivery, Health services, Resource-limited settings

## Abstract

**Background:**

Although Differentiated Service Delivery (DSD) for anti-retroviral therapy (ART) has been rolled-out nationally in several countries since World Health Organization (WHO)‘s landmark 2016 guidelines, there is little research evaluating post-implementation outcomes. The objective of this study was to explore patients’ and HIV service managers’ perspectives on barriers to implementation of Differentiated ART service delivery in Uganda.

**Methods:**

We employed a qualitative descriptive design involving 124 participants. Between April and June 2019 we conducted 76 qualitative interviews with national-level HIV program managers (*n* = 18), District Health Team leaders (*n* = 24), representatives of PEPFAR implementing organizations (11), ART clinic in-charges (23) in six purposively selected Uganda districts with a high HIV burden (Kampala, Luwero, Wakiso, Mbale, Budadiri, Bulambuli). Six focus group discussions (48 participants) were held with patients enrolled in DSD models in case-study districts. Data were analyzed by thematic approach as guided by a multi-level analytical framework: *Individual-level factors; Health-system factors; Community factors; and Context.*

**Results:**

Our data shows that multiple barriers have been encountered in DSD implementation. *Individual-level:* Individualized stigma and a fear of detachment from health facilities by stable patients enrolled in community-based models were reported as bottlenecks. Socio-economic status was reported to have an influence on patient selection of DSD models. *Health-system:* Insufficient training of health workers in DSD delivery and supply chain barriers to multi-month ART dispensing were identified as constraints. Patients perceived current selection of DSD models to be provider-intensive and not sufficiently patient-centred. *Community:* Community-level stigma and insufficient funding to providers to fully operationalize community drug pick-up points were identified as limitations. *Context:* Frequent changes in physical addresses among urban clients were reported to impede the running of patient groups of rotating ART refill pick-ups.

**Conclusion:**

This is one of the first multi-stakeholder evaluations of national DSD implementation in Uganda since initial roll-out in 2017. Multi-level interventions are needed to accelerate further DSD implementation in Uganda from *demand-side* (addressing HIV-related stigma, community engagement) and *supply-side* dimensions (strengthening ART supply chain capacities, increasing funding for community models and further DSD program design to improve patient-centeredness).

## Background

In Sub-Saharan Africa (SSA), it is common to find that HIV clinics are heavily congested with long patient queues. Long waiting times are typical at HIV clinics and health workers endure heavy workloads [1–7]. Due to the widespread overcrowding and the resource-constrained operational contexts of HIV clinics in SSA, innovations in HIV service delivery approaches have become imperative [1–7]. Differentiated Service Delivery (DSD) is one such innovation. DSD has been defined as *‘a client-centered approach that simplifies and adapts HIV services across the cascade, in ways that both serve the needs of people living with HIV better and reduce unnecessary burdens on the health system’* [2].

**Fig. 1:**
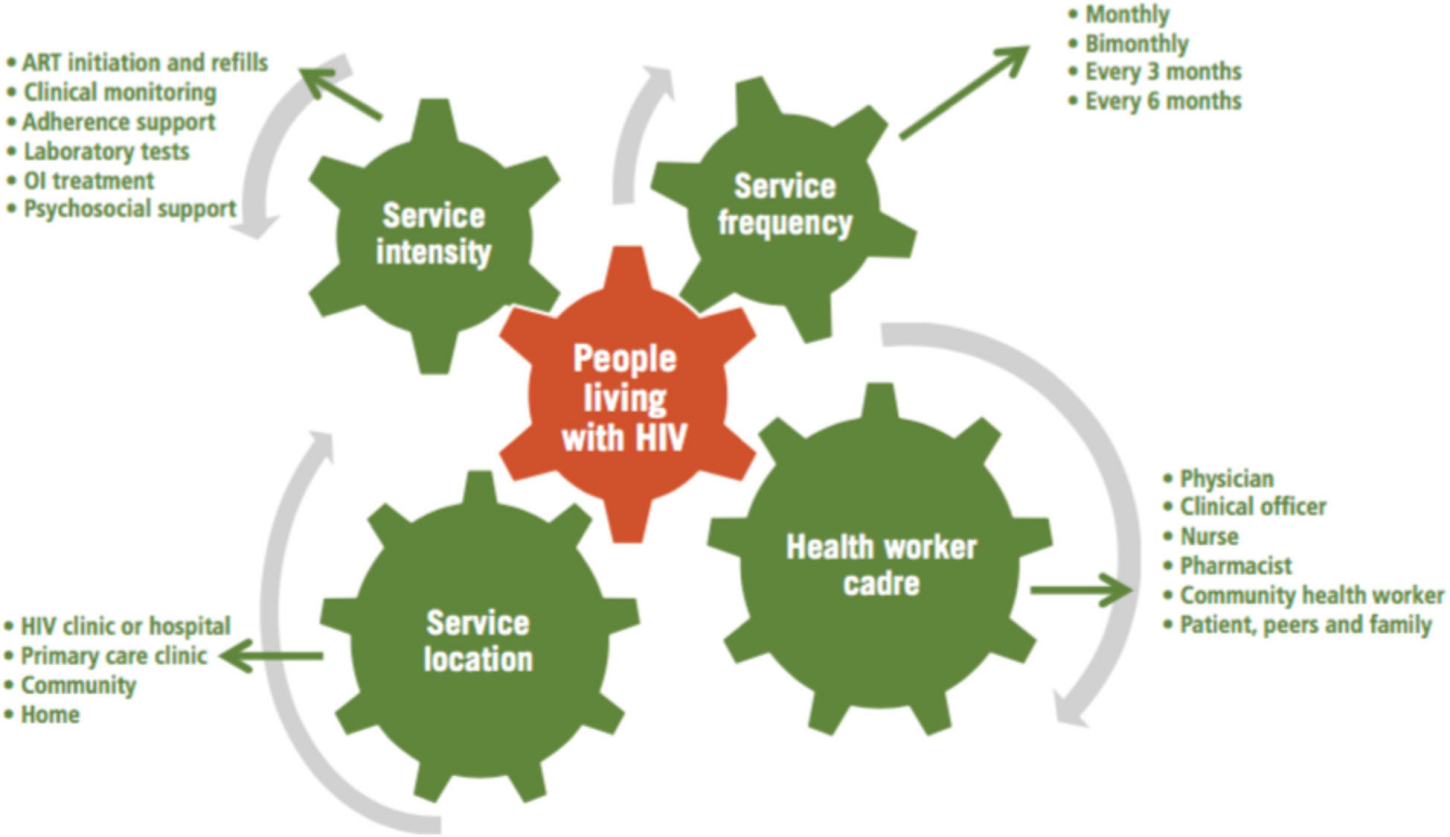
Duncombe et al.(2015)’s ‘Four levers to tailor or adapt HIV care to people’s needs

In 2016, DSD was endorsed by the World Health Organization (WHO) and leading global HIV donors such as Presidents’ Emergency Plan for AIDS Relief (PEPFAR) and The Global Fund to Fight AIDS, Tuberculosis and Malaria (The Global Fund) as a novel evidence-informed HIV service delivery approach that relieves pressure on over-burdened health systems in SSA [2]. In addition to improving health-system efficiencies, tailoring HIV care to the needs of individual clients as, opposed to ‘one-size-fits-all’ undifferentiated models of care, has been proven to improve patient outcomes and the quality of HIV care [1–4]. DSD embraces patient-centric approaches that seek to reduce unnecessary burdens of care on patients which results in savings in the time spent at facilities and the transport costs associated with more frequent visits to facilities [4]. As shown in Fig. 1, Duncombe and colleagues [7] posit that these innovative HIV service delivery alternatives constitute elements that entail a reduction in service intensity and frequency for stable patients, task shifting to non-clinical health worker cadre and changes in service location (such as co-opting community-based platforms).

Since 2017, several countries in SSA have been implementing DSD. Some of the countries rolling-out DSD nationally include Kenya, Uganda, Malawi and Zambia [8].

### National DSD implementation in Uganda

**Fig. 2:**
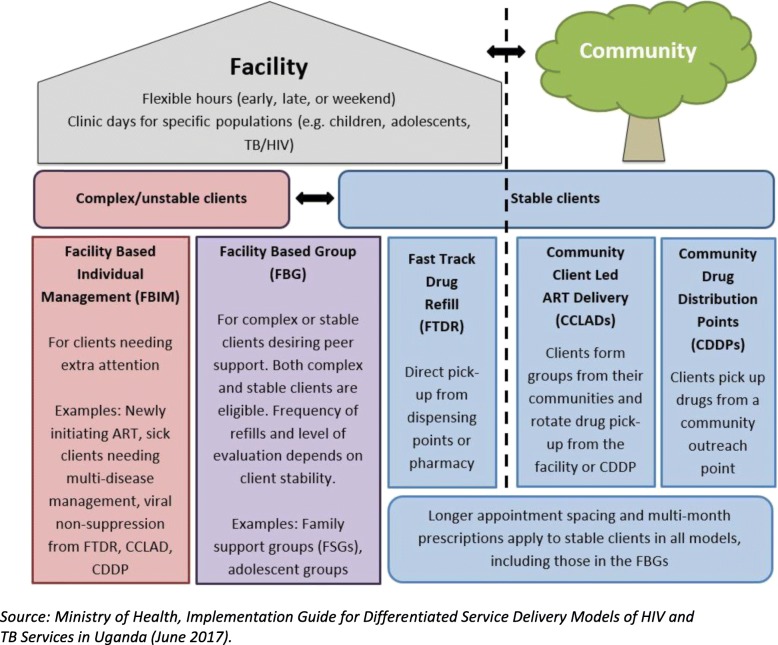
The five Differentiated Service Delivery models in implementation in Uganda. Source: Ministry of Health, Implementation Guide for Differentiated Service Delivery Models of HIV and TB Services in Uganda (June 2017)

In 2016, Uganda released updated national ART treatment guidelines providing for DSD in alignment with WHO treatment guidelines released the same year [9]. In 2017, PEPFAR, the predominant HIV donor in Uganda [6], included national DSD roll-out in its annual program targets for Uganda known as Country Operational Plan (COP 2017) [10]. As illustrated in Fig. 2, Uganda is currently implementing two broad categories of DSD models: i) Facility-based models and ii) Community-based models [9]. Figure 2 shows the five specific DSD models currently in implementation in Uganda. The facility-based models are three; i) Facility Based Individual Management (FBIM), ii) Facility Based Group (FBG) and iii) Fast-Track Drug Refill (FTDR). There are two community-based models; i) Community Drug Distribution Points (CDDP) and ii) Community Client-Led ART Delivery (CCLAD) [10]. Uganda is widely considered as a leader in DSD implementation because it was one of the first countries to provide for DSD in its national ART treatment guidelines as well in rolling it out nationally [11, 12]. Uganda therefore presents a unique opportunity of generating implementation research lessons with potential for broader application to other countries with a high HIV burden, especially those in resource-limited settings. By April 2019, the Ministry of Health and donors were training health workers in DSD delivery with almost 67% of health facilities covered across the country [12]. These on-going health worker trainings have also targeted select ‘expert’ patients or HIV client ‘peer leaders’ [12]. In Uganda, PEPFAR subsidiary local and international non-governmental organizations known as ‘implementing partners’ have also been mandated by PEPFAR to spearhead DSD roll-out at the sub-national level in geographic regions under their purview [10].

Most of the evidence on patient perspectives on Differentiated ART services has been drawn from clinical trials or controlled research settings [5, 7, 8, 13]. Although DSD has been rolled-out nationally in several countries since WHO’s landmark 2016 guidelines were released, there is a dearth of evidence on patient perspectives on HIV care under DSD in ‘real world’ settings or at the frontline level of service delivery [14–16]. A notable exception is a study conducted in Ghana [17]. However, that study only reported patient experiences of DSD from one tertiary hospital in Cape Coast Ghana. There is little research reporting post-implementation perspectives of patients and frontline health workers following national scale-up of DSD models in resource-limited settings [8].

Research reporting patient perspectives on DSD and their preferences is critical in further *program design* of DSD models which is still an evolving process [3, 8, 14–16]. Although DSD is an encompassing term that usually incorporates differentiated HIV testing and treatment, in this paper we focus on antiretroviral therapy (ART) delivery. ART constitutes one of the highest unit costs of HIV care and holds enormous promise of maximizing efficiency gains via DSD [4]. We particularly focus on patients deemed clinically stable on ART and are enrolled in DSD models through appointment spacing, multi-month ART dispensing, community drug pick-up points and patient support groups [3, 6]. Although national DSD implementation has been ongoing in Uganda since 2017, there have been little research evaluating implementation outcomes since initial roll-out. The objective of this study was to explore patients’ and HIV service managers’ perspectives on barriers to implementation of DSD for ART following a national scale-up program in Uganda.

## Methods

### Study design

This study employed a qualitative descriptive design aimed at understanding patients’ and HIV service managers’ perspectives on barriers to implementation of DSD for ART in Uganda. We aimed to explore the barriers to uptake of Differentiated ART service delivery models from the perspectives of participants within the context(s) underpinning their interface with the health system [18]. We utilized a case-study research design, which is recommended for in-depth investigation of complex phenomena [19].

### Analytical framework

This study is broadly guided by an analytical framework proposed by Levesque and colleagues [20] which is based on a systematic review that was conducted on factors influencing access to health care. This analytical framework proposes a multi-level lens incorporating both demand-side (individual-level factors such as knowledge, attitudes, and self-care practices) and supply-side factors (health-system factors such as availability of human resources and financing, enabling policies and physical infrastructure) in understanding influences on access to health care. The Levesque framework guided the study in two ways. We deliberately sought multi-stakeholder perspectives on DSD implementation which informed the diversity and range of the study participants selected. Secondly, the framework guided data analysis by providing an overarching deductive thematic framework in which to categorize our inductively-generated sub-themes presented in the results sections.

### Study sites and sample selection

In keeping with the multi-level analysis lens of the adopted analytical framework [20] of the study, multi-stakeholder participants were purposively selected to represent the *programmatic, provider and patient* perspectives on DSD implementation in Uganda at the national, sub-national and facility-levels (Table 1). Participants were drawn from national-level HIV program managers at the Ministry of Health’s AIDS Control Program (ACP), District Health Team leaders and representatives of PEPFAR implementing organizations. At the facility-level, we interviewed ART clinic in-charges and their staff as well as patients enrolled in Differentiated ART delivery models. We purposively selected health facilities in Uganda to achieve diversity with regard to a) ownership-type (public/private) b) level of care in the Ugandan health system and c) setting (rural/urban). The demographic characteristics of participating health facilities are shown in Table 2. Participating health facilities were drawn from the Central and Eastern regions of Uganda from six districts with a relatively high HIV burden and with a dense concentration of ART sites from which we could purposively sample (*Central*: Kampala, Wakiso, Luwero, *Eastern*: Mbale, Bulambuli, Manafwa).

**Table 1:** Category of participants (*n* = 124)

1. National-level HIV program managers	18
2. District health team leaders	24
3. PEPFAR ‘implementing partner’ representatives. (Local and international non-government organizations)	11
4. ART clinic in-charges and staff	23
5. Participants in patient focus group discussions	48

**Table 2:** Characteristics of participating health facilities

	PUB-01	PUB-02	PUB-03	PUB-04	PFP-01	PNFP-01
**Ownership-type**	Public	Public	Public	Public	For-profit	Not for profit
**Level of care**	Regional Referral Hospital	District Hospital	Sub-district health centre	Sub-district health centre	Clinic (HC III)	Health centre III
**Urban setting**	Urban	Urban	Peri-urban	Rural	Urban	Rural
**HIV services offered**	VCT, ART, PMCT	VCT, ART, PMCT	VCT, ART, PMCT	VCT, ART, PMCT	VCT, ART	VCT, ART

Key: *PMTCT* Prevention of mother to child transmission services, *VCT* Voluntary counselling and testing of HIV

We aimed to understand patient perspectives of HIV care under the various DSD models being implemented in Uganda. We sought to elicit patient perspectives on the challenges of enrolling in these novel ART delivery models through focus group discussions (FGDs) involving individuals enrolled in the same DSD models. As such, FGDs were deemed appropriate as they enabled a diversity of responses and allowed us to explore variations in patient experiences under the same DSD models [21]. For these FGDs, patients were eligible to participate if they had been enrolled in at least one of the DSD models currently on offer in Uganda (Fig. 2). Patients were enrolled if they had been accessing care in a DSD model for at least a year and voluntarily consented to eliciting experiences of HIV care under Differentiated ART delivery. We selected adults who were at least 18 years of age and were willing to offer written informed consent to participate in the study.

### Data collection

A topic guide was constructed around themes derived from the analytical framework adopted for the study [20]. These include: *i) Individual-level factors* e.g. knowledge, attitudes, self-care practices ii), *Health-system factors* e.g. Human resources, financing, policy iii) *Setting* e.g. influence of urban setting on choice of care as suggested by the Levesque framework [20]. This topic guide (supplementary file) was used to guide both our qualitative interviews and FGDs.

Overall, 23 semi-structured interviews (SSIs) were conducted with ART clinic in-charges and their staff across the case-study health facilities (Table 2). The aim of the interviews was to understand barriers to DSD implementation from a facility-level dimension and to explore health workers’ perspectives on the national scale-up of Differentiated ART services. Face-to face interviews were conducted in participants’ offices within the health facilities between April and June 2019. The interviews were conducted by the first author who holds a PhD in health systems and has an academic background in the social sciences and an expertise in qualitative research [22, 23]. The first author was assisted by three Research Assistants (RAs) experienced in qualitative health services research.

In addition, 53 semi-structured interviews (SSIs) were conducted with select participants with unique ‘insider’ knowledge on Uganda’s national DSD implementation planning and processes. These include national-level HIV program managers at Uganda’s Ministry of Health (*n* = 18), 24 District Health Team leaders (including District Health Officers or DHOs) and representatives of PEPFAR implementing organizations in case-study districts (*n* = 11) with unique knowledge of DSD implementation at sub-national level. The interviews were aimed at understanding the programming and policy dimensions of DSD implementation from national and sub-national perspectives of influential actors in the health system in Uganda whose actions influence the adoption of public health interventions. On average, these interviews lasted between 40 and 60 min.

In total, we conducted six focus groups discussions with each involving eight participants (Table 2). The focus group discussions were conducted on the designated ART clinic day at each of the case-study facilities when patients attended facilities for scheduled reviews. Participants were selected with the help of the ART clinic-charge based on a declared inclusion criterion. We explained the objectives of the research to all study participants including the nominated patients attending scheduled reviews at the ART clinics who were invited to participate on a voluntary basis. Written informed consent was obtained before focus groups could commence discussions. The focus groups were facilitated by the first author who was assisted by three RAs. The RAs took notes to ensure accuracy in transcription [21]. On average, the FGDs lasted 1 hour.

### Data capture and analysis

We followed the processes recommended for ensuring rigour in case-study and qualitative data analysis suggested by Gilson, L et al. (2011) (Table 3) [24]. We made audio recordings of all of the interviews and then transcribed each interview verbatim. In terms of data analysis procedures, we followed four major steps. However, this was a largely iterative process [25]. The first step involved *data familiarization* through multiple readings of interview transcripts by HZ, JR and JK [25]. The second step entailed *generating a coding framework.* Codes were inductively generated from the interview transcripts in a team-based process involving four authors (HZ, JR, JK, CK). The third stage was that of *abstracting the coded data into thematic categories.* The emergent inductive or data-driven codes were then grouped under a deductive thematic framework based on items selected from Levesque’s framework [20]*: i)* Individual-level factors ii), health-system factors iii) Community and iv) Contextual factors. Hence, our coding combined both inductive and deductive analysis approaches [26]. This process involved three authors (HZ, JR, and JK)*.* The fourth and final step was that of *Interpretation and overall synthesis.* A multi-stakeholder data validation workshop was conducted in June 2019 at which the initial study findings were presented. We invited eight national-level HIV program managers, 12 District Health Team leaders, 16 ART clinic in-charges and 11 patients ‘peer’ leaders to this one-day data validation workshop. The authors made a one-hour presentation of the study findings and invited comments and feedback from participants. Participants’ feedback informed the final analyses. All authors were involved in the overall synthesis of the study findings which were arrived at through a consensus process that resolved disagreements in interpretation [24, 25].

**Table 3:** Processes for ensuring rigor in case-study analysis adapted from Gilson et al. (2011)

PRINCIPLE	
**Prolonged engagement**	We spent 2–3 weeks at each of the six case-study facilities. Multiple on-site visits were spent engaging in informal discussions with ART clinic in-charges.
**Use of theory**	The analytical framework by Levesque et al. (2013) which proposes a multi-level perspective on factors affecting access to health care guided our analysis of the study findings.
**Case selection**	Six health facilities were purposefully selected in areas of Uganda with a relatively high HIV burden and a concentration of ART sites to enable purposive sampling.
**Sampling**	We aimed to have a sample that had appropriate representation of health facility demographics in Uganda with respect to a) setting (rural/urban), b) ownership-type (public, for-profit, not-for-profit), c) Level of care (tertiary, secondary, primary).
**Multiple methods**	Multiple methods were used including face-to-face interviews, focus group discussions (FGDs) and informal engagements with clinicians and ART Clinic in-charges.
**Triangulation**	Case descriptions were constructed based on triangulation across multiple data sources (Interviewee data and document review).
**Negative case analysis**	Emergent themes/ findings that contradicted initial assumptions were identified.
**Peer debriefing and support**	Data analysis at each of the four major stages involved a team-based process involving at least three authors.
**Respondent validation**	A multi-stakeholder data validation workshop was conducted at which the initial study findings were presented. Participant feedback informed the final analyses.

## Results

The findings emerging from this study are presented based on themes derived from the analytical framework advanced by Levesque and colleagues [20] described above. The broad themes proposed were individual-level, health-system, community and contextual factors.

### Individual-level factors

Focus group discussions with patients and interviews with HIV service managers revealed multiple individual-level barriers to enrollment in Differentiated ART delivery models in Uganda. These include internalized stigma, fears of detachment from the health system and limited patient literacy about Differentiated Service Delivery.

#### Internalized HIV-related stigma

Our findings show that internalized stigma is a fundamental barrier to enrollment in community-based DSD models due to patient fears of breach of confidentiality of their HIV sero-status which would be inadvertently disclosed to all members of a patient group to which an individual belongs. Individual fears of involuntary disclosure of HIV status to peers was frequently cited as an impediment to enrollment in DSD models across our focus groups with patients and interviews with health workers. As one patient said:

*‘Patients don’t want to join CCLAD groups because they say ‘so and so will get to know that I have HIV’. So, the lines are still long at my hospital because people still live in fear to come out and join patient groups for picking their medicines because they think if you take for him ARVs then you will tell somebody else who was not aware of their HIV status. So people still have that fear’ Patient, FGD, PUB-01.*

#### Fear of detachment from the health-system

Numerous patients who were enrolled in community-based DSD models expressed a fear of detachment from the formal health-system. The majority of these recipients of care were deemed stable on ART and hence did not have a clinical need to make monthly visits to HIV clinics. Several patients in the FGDs described deriving psychosocial support in regular face-to-face interactions with health workers. Some patients also expressed personal attachment to individual health workers who they had grown accustomed to meeting monthly for scheduled reviews. Patients frequently expressed fears that prolonged periods without being seen by health workers would imply inability to access comprehensive care including in the event of opportunistic infections such as Tuberculosis (TB).

*‘When the clients are given drugs for three months, one may get an attack like TB or another opportunistic infection so it may take long for health workers to discover. When you are in the CDDP groups you feel you are no longer part of the health system. Sometimes you sit there and think, they took us to the community to die from there’ Patient FGD, PNFP-01.*

Overall, our findings from FGDs reveal that the majority of patients appeared to prefer facility-based models to community-based DSD models. This notion seemed to hold even among health workers. Many health workers perceived FTDR to be the most practical DSD model to implement hence patient enrollments were reported to be skewed as such. Community-based DSD models, especially Community drug pick points, were described as costly to implement as they required fuel for transporting health workers into communities to monitor patients in this model of care, preparing pre-packaged ART medicines and finding suitable physical infrastructure to designate as community drug pick-up points in remote, rural settings. Hence health-system constraints appeared to interact with individual-level choices in influencing patient uptake of (especially) community-based DSD models.

*‘The enrollment in fast-track refill models is high which is good because it is the easiest to implement at the facility level in my opinion. The guidelines are very clear, two viral loads and you are stable, no problems, it’s easy’ ART clinic in-charge, PUB-01.*

The national-level HIV program managers reported national statistics on patient enrollment in DSD models that appear to corroborate our qualitative findings.

*‘Currently, facility-based models account for the biggest proportion of enrollment in DSD models. Fast- Track Drug Refills stand at 38%, Facility-Based Groups are at 9% and Community Client-Led ART Delivery are at 7%. Nevertheless, the Ministry of Health is very optimistic about seeing more utilization of community models’ National-level, HIV program manager.*

Contrary to what has been reported in the literature, a number of patients discounted the advantages associated with reduced frequency of visits to facilities such as reported savings in time and transport costs. Health workers reported that although patients are frequently sensitized about the advantages of enrolling in less-intensive DSD models during their visits to the facilities for clinical reviews, some patients expressed a willingness to meet the costs of frequent visits to facilities especially those in urban settings.

*‘Who told you I don’t have transport money to come here (at health facility) every month? For me I am prepared and ready to pay my 10,000 Uganda shillings ($ 2.74) every month to come here to pick my drugs’ Patient FGD, PUB-02.*

*‘When it comes to DSD we are not on the same page with patients. Although DSD confers several advantages to patients such as savings in time spent at facilities and a reduction in transport costs incurred in seeking care, patients have other considerations which we have found to be contrary to our expectations’ ART clinic in-charge, PNFP-01.*

Health workers at two participating facilities (PUB-01, PUB-02) reported increasing cases of patients requesting self-referrals to especially private health facilities (many of which had not yet started implementing Differentiated ART delivery) on account of their reluctance to join patient groups especially involuntary ones initiated by health workers in some facilities.

#### Low patient literacy of DSD models

Low patient literacy of DSD models was a recurring theme across our interviews with health workers and in our FGDs with patients. It emerged that patients had not been sufficiently sensitized on the merits of enrollment in DSD models and there remained demand-side gaps in knowledge and awareness about DSD.

*‘As a client in Kampala, I rarely hear mention of DSD at my facility. Even my fellow clients don’t know about DSD. That is a fact. As a peer-leader, if I tell them about DSD they have not seen it in practice’ Patient FGD, PUB-03.*

National-level HIV program managers reported that there was a section of patients across Uganda who had not yet been reached by national DSD sensitization drives and community engagement efforts across the country which were reported to be on-going. Our interviews revealed that where DSD community-engagement drives had been conducted in Uganda they had targeted only a section of health workers and patient ‘peer-leaders’ who had not yet widely disseminated to the broader base of patients at health facilities.

*‘Some of the patients have not heard about DSD, but this not surprising because we have not yet reached 100% of health facilities. Even in the Central Region, not all facilities have been trained. When we do facility-based trainings, the patients that we actually reach are the peer leaders’ National-level HIV program manager.*

The national-level HIV program managers reported that increased health education talks for patients had been followed by increased DSD uptake including community-based models.

*‘Facilities where sensitization has happened, you see that the uptake not only for the facility-based but even the community models goes up because then patients appreciate why they should actually join’ National-level HIV program manager.*

One emergent finding from our interviews with health workers was that patient preference of DSD models was partly influenced by their socio-economic status. Specifically, health workers reported observing trends suggesting that some urban clients with relatively high income preferred facility-based individual models due to a perceived higher need of privacy and confidentiality. Lower-income individuals especially those who couldn’t afford monthly transport costs of about 10,000 Uganda shillings (US$ 2.7) were said to prefer community-based models such as CDDP because they enabled them to make savings in transport costs.

*‘What we are seeing is that patients who are better off (financially) decline joining community groups such as CCLAD and CDDP because they crave privacy and prefer to receive individualized care at the health facility. But the reverse is true of our poorer and rural clients who prefer community models such as CDDP which reduce their transport costs’ ART clinic in-charge, PUB-02.*

### Health-system factors

#### Health worker competence in DSD delivery

Health worker competence in DSD was revealed as a bottleneck in service implementation in our sample of health facilities. National-level HIV program managers revealed that some health facilities had health workers who had not yet been trained in DSD delivery while for those facilities which had been reached by DSD training programs of the Ministry of Health, only a proportion of their health workers in the ART clinics had been trained in DSD delivery. Interviews with national-level HIV program managers revealed that 67% of health facilities across Uganda had been covered by the national program on health worker training in DSD service delivery.

*‘Currently, we have trained health workers in 1,200 (out of 1,800) health facilities providing ART in Uganda. They have been engaged and trained on DSD implementation. Only 600 facilities are yet to be covered’. National-level HIV program manager.*

Our findings in Eastern Uganda suggest that health worker trainings in DSD delivery did not necessarily translate into implementation. This was particularly the case in lower-level health facilities especially those at the county (Health Centre IVs) and sub-county (Health center IIIs) levels. Multiple implementation barriers were cited that include insufficient funding for running community-based DSD models.

#### Frequent stock-outs

Frequent ART medicines stock-outs were highlighted as a recurring bottleneck in implementing DSD models particularly the FTDR and CDDP models. Across our interviews with health workers and focus groups with patients it was revealed that clients, in some case-study facilities, were getting one-month or even a two-week supply of anti-retrovirals (ARVs) owing to frequent stock-outs. Participants confirmed that there were country-wide ARVs stock-outs in the last quarter of 2018 which impeded multi-month refills which are a cornerstone of Differentiated ART delivery.

*‘Drug stock outs are a big challenge. We had stock-outs in the last quarter of 2018 and the first quarter of 2019. Those of us on fast-track drug refills, instead of being given a three-month supply, we were getting one month and even two weeks at one point. So, how will we sustain the model?’ Patient FGD, PUB, 002.*

#### DSD not implemented in lower health facilities

We observed variations in DSD implementation by level of care in the Ugandan health system. In our sample of health facilities from Eastern Uganda, DSD implementation was reported to have commenced at the tertiary-level (regional and district hospitals). However, participating lower-level health centers (sub-district and sub-county health centers) indicated they hadn’t yet implemented DSD.

*‘DSD is being rolled out at the level of tertiary hospitals and not yet at lower level health centers. Although some facilities have been trained in DSD services, they have not gone ahead to implement. The training was done but the implementation has not yet taken place because of so many issues’ ART clinic in-charge, PFP-01.*

In our FGDs at sub-district public facilities in Eastern Uganda, patients indicated that some of their peers had heard about DSD although several of them had not yet been enrolled into DSD models. We found a handful of centers of excellence in HIV care such as The AIDS Support Organization (TASO) where a majority of their patients were enrolled in DSD models.

*‘DSD is not a totally a new concept. It was named DSD but you will notice that in Uganda, we had already done differentiation. People were coming after every two months, after three months and in some places, like the centers of excellence like TASO. Actually, most of our learning around DSD was from TASO*, *they had already initiated the community drug distribution points’ National-level HIV program manager.*

#### “Unstable’ as a stigmatizing label

Patients perceived the terms used in DSD nomenclature of *‘unstable’* and *‘stable’* as provider stigma. Although the terms ‘unstable’ or ‘stable’ denote clinical assessment of whether patients are doing well on treatment or not and therefore their eligibility for the various DSD models, patients expressed disapproval of the use of the term *‘unstable’*.

*‘Using the term ‘unstable’ and ‘stable’, to me and I think to some of us, that language is stigmatizing. If you tell me that John you are ‘unstable’, I will feel down. I feel that is unfair to m*e’ *Patient FGD, PUB-01.*

Interviews with health workers revealed that prior to the introduction of DSD nomenclature in Uganda’s national ART guidelines of 2016, ART-providing organizations had devised more acceptable in-house terms to refer to ‘stable’ and ‘unstable’ patients. This included the use of colors to indicate a patient’s clinical status such as those suppressing and those not suppressing. Health workers maintained that DSD was not an entirely new approach in Uganda and that they had originally devised terms that were more patient-sensitive before the introduction of new nomenclature following country-wide DSD roll-out.

*‘Where I get care they call it a ‘pink card’. When you get a pink card it means you are stable, you do not need to see a doctor all the time and people really strive to earn that card. They even call their doctor and say ‘doctor, I have got a pink card and I am so happy’. It is like a graduation’ Patient FGD, PUB-003.*

*‘In Uganda, these are not the words we use because when you go to Buganda (central Uganda), they have simplified the ‘stable’ and ‘unstable’ by using local language alternatives. You get it? But our nomenclature in English of defining this person who is not suppressing is wanting. I understand and appreciate patient concerns’ National-level HIV program manager.*

#### DSD is not client-centered

Patients and health workers concurred in relaying the notion that although DSD was intended to be a client-centered, current DSD delivery especially decisions on assignment of DSD models were intensely provider-directed and patients did not meaningfully participate in making decisions regarding which DSD models in which to be enrolled. Although clinical criteria are paramount in patient differentiation, focus groups with patients revealed that their individual preferences were rarely put into consideration in assessing their readiness for a particular DSD model.

*‘Patients are just told that “you, you will be getting drugs from your community”. We are told that it is client –centred but is it? Clients should be involved in making decisions about their care’ Patient FGD.*

National-level HIV program managers revealed a need to engender client-centeredness in the curricula of the on-going health worker DSD trainings across Uganda and to the need to provide opportunity for the participation of patients in DSD program design and the further refinement of these models.

‘*We are trying to build the capacity of health workers in letting them know that groups that are self-formed are groups that are going to last. We have seen instances where the health workers go ahead and prescribe and two months down the road, everybody they put in that group is no longer there. Because they practically push them there’ National-level HIV program manager.*

#### Clash between DSD and tuberculosis appointment spacing

An important finding of this study is that patients enrolled in DSD models such as those enrolled in the FTDR model or those receiving multi-month ART refills but who were also on TB management, were still expected to make monthly visits to the health facility regardless of whether they were stable on both ART and TB. Both health workers and patients perceived this as a practice that undermines the intended benefits of differentiated care of reducing burdens on patients who are clinically stable.

*‘TB is one of the new areas that is coming up. How do we do differentiation for TB services? For example, you are giving preventive therapy of isoniazid for one month and yet this person who is stable on ART gets their review every 6 months, how do we reconcile these two?’ ART clinic in-charge, PUB-03.*

A few of the patients in our focus groups, particularly older HIV patients with co-morbidities especially Non-Communicable Diseases (NCDs) such as hypertension and diabetes reported that their NCDs conditions were being managed separately from their HIV care needs and the benefits of reducing the frequency of clinic visits were not being realized for them despite DSD implementation.

*‘I am 63 years old and I have been on ART for eleven years. Last year during a routine check they found I had both pressure (hypertension) and sugar (diabetes). Now, I have to visit the Diabetes clinic every month yet I am stable on ART and visit the HIV clinic once every three months’. Patient FGD, PUB-01.*

*‘We have clients who are in the age groups of 50 and above, most of them due to cohort ageing, have NCDs and it gets difficult to have these people get into the drug refill programs the more you have a mature cohort the more you have other problems coming up’ Health worker, PUB-01.*

### Community-level factors

National-level HIV program managers reported that patient enrollment in community-based DSD models across the country stood at between 5 and 7%. Participants reported that community DSD models were beset by multiple constraints ranging from HIV-related stigma to insufficient funding for operationalizing these models across Uganda.

*‘Enrollment in community-based models is at about 5-7%. We need to see more involvement of stable patients in community-based models which is where everybody should be comfortable to avoid congesting health facilities when they are well’* National-level HIV program manager.

#### Community-level stigma

Across our interviews with health workers and focus groups with patients, stigma within communities stood out as a critical barrier to realizing the full potential of DSD in relieving pressure on over-burdened health facilities.

*‘Community models are not very popular with clients. One of the reasons cited is stigma. Patients in many health facilities prefer to receive care at the health facilities because they are afraid of stigma from other community members*. *You know when you join a CCLAD group of ten people, all those ten people will now know your HIV status. And these are people who live in your neighborhood, in your village. Stigma is really a big challenge’.* ART clinic in-charge, PUB-01.

*‘As we implement these models, it is incumbent upon us to regard stigma as a key issue. Stigma is a key challenge which is still exists in our communities. This is what I have found during the on-site supervisions of DSD that I have conducted. Patients prefer to stay at the facility because of stigma within the community’ National-level HIV program manager.*

#### Implementation challenges associated with community-based DSD models

Health workers reported that several challenges had been encountered in implementing community-based DSD models. The CDDP model was frequently described by ART clinic in-charges as one that required substantial financial and human resources to implement. The challenges elicited include the need for vehicles and fuel to transport health workers into communities to deliver ART refills. The need for health worker monetary allowances during community visits and the difficulty in finding suitable physical infrastructure in rural settings to designate as outreach points for ART refills. With regard to the CCLAD model, the financial costs of off-site monitoring of stable patients within communities was identified. The difficulty of finding competent and literate leaders of CCLAD groups within client populations was frequently raised.

*‘The problem we face is that most CCLAD groups are failing. You find that you need to be 3-6 members in a group that resides in the same location. You may find that all the six members are illiterate. They can’t read, they can’t write and none is willing to take lead and when you are a leader, you need to do some documentation. So that has been a challenge for us’ Patient FGD, PUB-04.*

Group leaders of patient groups expressed difficulty in sustaining transport costs to facilities to pick drugs on behalf of their colleagues. Although picking drugs from the facility was meant to be a rotating responsibility among group members, it was common to find that, in many of the groups, the burden was frequently shouldered by a single member.

*‘Most of our clients come from hilly places they spend about 10,000 shillings ($ 2.7) to and from. So, I told them, if we form a group of 10 members, instead of each one of us spending 10,000 you can give 10,000 to one person we have selected to go pick our medicines. They accepted but I remain with that transport burden alone. My income is very little yet I have to support this group’ Patient FGD, PUB-04.*

Leaders of CCLAD groups who pick drugs from health facilities on behalf of their members reported difficulty in identifying ART refill packages for each of their individual members. Delivering incorrect drug packages to their members was reported to happen in some instances as narrated in the quote below:

*‘There are challenges in identifying individual drugs for members of the CCLAD patient group. I can be a group leader delivering drugs to other clients but you realize someone says ‘they have packed for so and so different ARVs, yet he takes a different regimen’* Patient FGD, PNFP-01.

Health workers decried the additional workload involved in packaging and labelling ARVs drug packages for each individual member in models involving decentralization of drug delivery to communities. In high-volume facilities, the number of patient (CCLAD) groups were said to be as many as 40. Since each of these patient groups had an average membership of six members, the burden of preparing ART refill packages had increased workloads. This was a notion frequently raised by health workers and HIV service managers.

*‘Labelling drug packages for those on multi-month scripts is a headache. You need to indicate on the bottles that these drugs are for month number one, and this is month is for month number two and three. So, if you have 6,000 clients in DSD models that shows just how much work you have to put in packing drugs and correctly labelling them for each and every individual’ ART clinic in-charge, PUB-01.*

#### Insufficient funding for implementing community models

A common refrain from the health workers was the insufficient funding for operationalizing community-based DSD models. The CDDP model was frequently cited as an example of community-based models that require substantial funding to implement. Health workers mentioned the need for constant fuel for health workers to travel outside of the health facilities into the communities to monitor patients, transport for ferrying ART refills into communities and the difficulty of finding suitable physical space in remote communities to designate as drug pick-up points. A concern that was frequently raised by health workers was that of the sustainability of community-based DSD models, such as CDDP which are currently heavily donor-dependent. National DSD scale-up in Uganda has depended substantially on PEPFAR funding since initial roll-out in 2017. Participants perceived facility-based DSD models as more sustainable in the event of loss of donor support. We observed a widely-held perception that community DSD models were expensive to implement and unsustainable without international assistance.

*‘I think that the best DSD models should remain the facility-based ones because it is not sustainable going into these communities. You are able to deliver these medicines now just because there is donor funding but time is going to come when there is no funding’ ART clinic in-charge, PUB-02.*

### Contextual factors

Overall, our interviews with health workers and FGDs with patients appeared to relay the notion that setting was influential on the uptake of individual DSD models. In our sample of health facilities, patients hailing from urban settings expressed preference for facility-based individual models over community-based models.

*‘There are certain unique issues in urban areas like in Kampala (capital city). People do not want to form groups. They just want to go to the facility, get their drugs and go home, or go to a point somewhere to get their drugs and then go home’ PEPFAR Implementing organization representative.*

#### Mobility among urban clients

Health workers of case-study facilities located in urban settings reported that patients frequently changed residential addresses. The dynamic nature of their urban patients was said to impede the smooth running of self-formed patient groups (such as CCLAD) which require stable populations that reside in the same physical location for them to thrive.

*‘How do you constitute the groups especially in Kampala (capital city) which is dynamic because people keep moving and frequently change residential addresses? It has been difficult’. ART clinic in-charge, PUB-01.*

Our focus groups revealed that HIV-related stigma was especially pronounced in urban settings and patients in case-study facilities located in urban areas expressed a reluctance to form groups with peers who lived in close physical proximity. This was raised as one of reasons why patients formed inconvenient groups of individuals (CCLAD) living in disparate locations for fear of breach of confidentiality of their HIV status with recipients of care living in the same neighborhood.

*‘The CCLAD approach is not working as well as anticipated due to stigma especially in urban areas. Patients form inconvenient groups with people living in different areas because of stigma. You will find groups where one client is from Kawempe (North of the capital) forming with a client from Nakawa (East of the capital) and may be Makindye (South of the capital) and they will choose a place in the city center where they will receive their drugs’ Representative of PEPFAR implementing organization.*

Participants from a case-study facility in the Ugandan capital Kampala reported that HIV-related stigma impeded the running of community ART refill pick-up points and they were compelled to devise alternative distribution points through private retail pharmacy networks in Kampala.

*‘What we did in Kampala in collaboration with the National Drug Authority is to just make patients pick their medicines from a nearby (retail) pharmacy because they are not interested in forming groups, they are not interested in doing anything else so they just go to a nearby pharmacy, pick their drugs and in ten minutes they are out’ PEPFAR Implementing organization representative.*

## Discussion

Although several countries in Sub-Saharan Africa have been implementing countrywide DSD roll-out since 2017, there is a dearth of evidence on early program implementation outcomes [27]. This is one of the first multi-stakeholder evaluations of national DSD implementation in Uganda since its initial roll-out in 2017. In this study, participants reported that they had encountered multiple implementation barriers in the adoption of DSD from both a demand-side and supply-side dimension of the health system [20]. Specifically, from the demand-side perspective, barriers to enrollment in DSD models relate to individualized stigma and a fear of detachment from the formal health-system for stable patients enrolled in community-based models. In this study, health workers reported that lower-income and rural patients prefer community-based DSD models while urban and financially wealthier patients tended to prefer facility-based models due to a higher expressed need for privacy and confidentiality. From a supply-side perspective, participants raised multiple logistical complexities and implementation challenges. These include frequent stock-outs which undermined multi-month ART prescribing and insufficient funding for operationalizing community DSD models such as outreach drug pick-up points. Patients perceived the assignment of DSD models as not sufficiently patient-centred.

Our study illuminates the diverse preferences of patients and underscores the notion that there is no ‘one size fits all’ DSD model due to the varying needs and characteristics of patients which are influenced by a variety of factors, which, in this study, we found, include socio-economic status and the rural-urban dynamics of setting. A study from South Africa published in 2019 [28] found that that community-based DSD models did not work for everyone. There has been broad acknowledgement in the literature that with regard to differentiated HIV care, patients’ preferences are complex and that further research is warranted to better understand this phenomenon [2–4, 8, 14, 29–31]. We call for future research to explore whether socio-economic status and rural-urban setting have a bearing on patient choice of DSD models especially if such studies use large samples of patients.

Contrary to what has been reported in the literature about the benefits realized by patients such as savings in transport costs and time spent at facilities via DSD when compared to more intensive undifferentiated care models [13, 17, 28, 32] our findings suggest that the picture is more complex and patients have other considerations in the models of HIV care they prefer. For instance, some patients in our study sample, preferred frequent clinic visits with a few expressing a willingness to spend money and time despite awareness of the benefits of DSD in reducing the burden of treatment. We found that stable patients enrolled in community models feared a detachment from health facilities and felt that they would not receive comprehensive care and treatment in the event of opportunistic infections such as tuberculosis if they remained in community-based models owing to their being categorized as ‘stable’. Previous studies have highlighted the psycho-social satisfaction patients derive from engaging with health workers on a regular basis [33]. Adjetey and colleagues [17] in a study in Ghana report that patients preferred facility-based HIV services to community-based care even after the government there had invested considerably in the latter models.

From a supply-side or health-system dimension, our study highlights the multiple implementation challenges encountered in DSD roll-out across Uganda. The frequent stock-out of ART medicines stood out in participant discourses which suggests that Uganda’s current pharmaceutical supply chain architecture is not yet attuned to the new levels of performance demanded by DSD implementation such as multi-month ART dispensing. At the level of human resources for health, we found that health worker trainings in DSD delivery are still on-going across the country which impedes DSD coverage rates at health facilities in Uganda and poses questions relating to the quality of DSD services currently on offer in Uganda. For instance, several patients perceived current DSD delivery in case-study facilities not to be sufficiently patient-centred. In this study, we found a widely held perception among health workers, and even national-level HIV program managers, that some community-based models, particularly the CDDP model, were expensive to implement and that current funding levels were insufficient to fully and widely operationalize them [34, 35]. This calls for further research around the cost effectiveness of select DSD models using data from Uganda or similar settings. A study by Sharer and colleagues [28] reports human resources and financing challenges in national DSD implementation in South Africa. There has been broad acknowledgment in the literature of health-system capacity constraints in moving DSD from pilot to scale in resource-constrained settings [3, 7, 8, 14, 36].

### Policy and programming implications of our study

Our study has a number of policy and programming implications for the Uganda government and donors. We found that HIV-related stigma was a fundamental barrier to patient enrollment in community-based models of care. Our findings suggest that there is sub-optimal implementation of community models and that the full potential of DSD in decongesting clinics and reducing workloads has not yet been realized in participating facilities. For donors such as PEPFAR, which funds implementing organizations at the sub-national level in Uganda, we found variations in DSD coverage in the districts we sampled which may be suggestive of a need for geographic prioritization in DSD scale-up efforts through pivoting to geographic sub-regions that are lagging behind. Devising stigma-reduction interventions through counselling, health education campaigns and sustained community engagement in Uganda are critical to optimizing the efficiencies promised by differentiating HIV care and treatment [36–38]. Some patients in our study expressed dissatisfaction with the level of *patient-centeredness* in current DSD delivery which may point to the need to improve patient participation in decision making in HIV care. This calls for increased engagement of health workers through trainings aimed at enhancing patient-centered HIV care as well as further research in DSD program design to enhance this notion. Providers called for increased funding from Uganda government and donors to facilitate full operationalization of community-based models such as facilitation for designating outreach sites for delivering drugs to stable patients [34]. Strengthening Uganda’s pharmaceutical supply chain system to align with the new performance demands imposed by multi-month scripting is a priority that requires re-orienting policy and business process re-engineering especially by the National Medical Stores (NMS) and other actors along the ART medicines supply chain. Overall, our study suggests that national DSD implementation is unlikely to follow a linear or ‘straight’ path as laid out in Uganda’s national DSD implementation guide but will require an iterative and dynamic posture that conforms more to the characteristics of a ‘complex adaptive system’ [39] approach in responding to the multiple logistical complexities and implementation barriers. MacGregor and colleagues [40], in a study in South Africa, found that moving ART adherence clubs from pilot to scale in the South African health system was wrought with ‘complexities’. They note that differentiated HIV care innovations such as ART adherence clubs on a ‘small scale’ appear ‘excellent’ but when implemented on a ‘large scale’ challenges emerge.

### Study implications for countries with similar setting as Uganda

Our study has implementation research lessons for other countries in resource-limited settings rolling out differentiated ART models. Our study findings underscore the enormity of *demand-side* barriers to enrollment in Differentiated models of care which are often under-explored in DSD scale-up efforts. Psycho-social barriers such as HIV-related stigma are often under-explored in national scale-up efforts with an over-emphasis on programmatic scale-up targets in spite of this critical bottleneck [31]. In a study in South Africa, Hanrahan and colleagues [30] conclude that “we urge caution in assuming that the effectiveness of clinic-based interventions will carry over to community settings, without a better understanding of patient-level factors associated with successful retention in care”. A study in Malawi found that frequent changes in the residential addresses of individual members of patient adherence support groups impeded retention in community-based care platforms [38]. On the ‘supply side’ dimension, our findings underscore the importance of strengthening ART medicines supply chains and policy changes to facilitate the decentralization of dispensing into communities [8, 36]. There is need for deepening the capacity of suppliers for managing the dramatic increase in demand or the sheer volumes of ART medicines to be dispensed occasioned by DSD implementations such as multi-month scripting [7, 41]. Our findings point to the need for harmonizing appointment spacing in HIV care with the management of other co-morbidities such as diabetes and hypertension [42, 43]. We found that older patients (50 years and above) who had well-controlled sugar levels or blood pressure and were also stable on ART were still required to make monthly clinic visits regardless of the DSD provision of 3-monthly visits. This calls for the integrated management of HIV and other co-morbidities that is becoming increasingly important priority due to ageing cohorts of clients and the need to revisit treatment guidelines even in non-HIV services [42–44].

An important finding of this study is that socio-economic status was perceived to have an influence on patient selection of DSD models. We found that patients who couldn’t raise the 10,000 Uganda shillings (US$ 2.7) average direct cost of visiting facilities preferred community drug pick-up points and peer support in sharing the transport costs associated with picking ART refills from facilities. On the other hand, a select number of urban patients expressed a preference for facility-based individualized care and a willingness to meet the costs associated with more frequent visits to facilities. There is some support in the literature for this notion of heterogeneity in patient preferences [16, 28, 31, 45, 46]. This may call for planning and programming that puts the complex and diverse preferences of patients into consideration in the quest to scale-up differentiated models of HIV care.

### Limitations

Our study has a number of limitations that we wish to acknowledge. We utilized a case-study approach of six health facilities in Uganda. Although this enabled us to have an in-depth insight into DSD implementation at the facility and community-levels, our study findings may not be fully generalizable to all HIV service delivery settings across Uganda [19].

### Strengths

This study had several strengths which include a multi-stakeholder lens into national DSD implementation in Uganda incorporating actors at the programming, provider, policy and patient levels [18, 20]. Additionally, we elicited national and sub-national level insights thus providing a more rounded perspective on the *early* implementation experiences of national DSD scale-up in Uganda.

## Conclusion

This is one of the first multi-stakeholder evaluations of national DSD implementation in Uganda since its initial roll-out in 2017. Multi-level interventions are needed to accelerate further DSD implementation in Uganda from both a *demand-side* perspective such as addressing HIV-related stigma and community engagement to improve DSD uptake and a *supply-side* dimension such as strengthening ART supply chain capacities, increasing funding for community DSD models and improving DSD program design to embrace patient-centeredness.

## Supplementary information

**Additional file 1.** Topic guide for focus group discussions with patients enrolled in DSD models.

## Data Availability

The datasets generated during and/or analyzed during the current study are not publicly available due to ethical reasons but are available from the corresponding author on reasonable request.

## Abbreviations

AIDS: Acquired Immune Deficiency Syndrome
ART: Anti-retroviral therapy
ARVs: Anti-retrovirals
CCLAD: Community Client-Led ART Delivery
CDDP: Community Drug Distribution Points
DSD: Differentiated Service Delivery
FBIM: Facility Based Individual Management
FBG: Facility Based Group
FTDR: Fast-Track Drug Refill
MOH: Ministry of Health
PEPFAR: The Presidents’ Emergency Plan for AIDS Relief
RA: Research Assistant
SSA: Sub-Saharan Africa
WHO: World Health Organization
