# Supplementary material for: Understanding implementation barriers in the national scale-up of differentiated ART delivery in Uganda
Source: BMC Health Serv Res. 2020 Mar 17;20:222. doi: 10.1186/s12913-020-5069-y (PMC7077133; doi:10.1186/s12913-020-5069-y)
Supplement: Supplementary file 1 — Additional file 1. Topic guide for focus group discussions with patients enrolled in DSD models. [file 12913_2020_5069_MOESM1_ESM.docx]

Patient topic guide for FGD with recipients of care in DSD models

**Instructions:**

- The FGD should be conducted in quiet setting.
- The FGD should be conducted by a facilitator assisted by a note taker who will take notes during the FGD.
- An audio recorder should be used to record the proceedings.
- Before commencing discussion patients need to voluntarily offer written consent
- Check informed consent: 🞎 YES 🞎 NO

INTERVIEW QUESTIONS.

1.       Please briefly tell me about yourself and how long you have been on ART.

2.       Before the introduction of DSD models, how frequently did you attend this facility for scheduled reviews?

3.       When and how did you come to be enrolled in a DSD model?

***Individual-level factors***

4.       Please briefly tell me about the DSD model you are currently enrolled. How did you come to be enrolled in it?

5.       What advantages do you find in being in your current DSD model?

(*Probes: i) Savings in time ii) savings in transport costs iii) benefits of less time away from work iv) reduced congestion*

6.       What challenges, if any, are you experiencing in the DSD model in which you are currently enrolled?

*(Probes: individual DSD models i) CCLAD ii) Fast-track refill (stock-outs) iii) FBIM iv) CDDP*

7.       If you were to choose a DSD model that you prefer which would this be? Why do you prefer this particular model?

***Health-system factors***

8.      How satisfied are you with the competence of health workers in providing DSD services to you?

 9. What is your comment on the level of patient awareness and sensitization on DSD services?

10. Differentiated Service Delivery is meant to be *patient-centered.* What is your comment on the extent to which current DSD implementation reflects your personal choices and preferences?

11. How have patients been involved in the development and implementation of DSD in your facility or community, if at all?

*(Probes: i) development of treatment guidelines ii) SOPs iii) improving DSD models)*

12. What is your comment on your health facility’s ability to provide you with 3-monthly ART refills? Have you experienced any stock-outs?

***Community factors***

13.  In your opinion, why has the uptake of community-based DSD models not been as strong as intended?

14.   What barriers to do you see to increased uptake of community-based DSD models? (*Probes patient literacy of DSD models, demand-creation campaigns prospects, stigma).*

***Setting***

15.   Do you know any of your fellow patients who have declined to enroll into some DSD models or DSD altogether? Why did they opt against being enrolled into DSD model(s)?

16.   Is there anything you want to tell us about DSD that we have not talked about?
